# Genetic Characteristics and Metabolic Interactions between *Pseudocercospora fijiensis* and Banana: Progress toward Controlling Black Sigatoka

**DOI:** 10.3390/plants11070948

**Published:** 2022-03-31

**Authors:** Roslyn D. Noar, Elizabeth Thomas, Margaret E. Daub

**Affiliations:** 1 NSF Center for Integrated Pest Management, North Carolina State University, Raleigh, NC 27606, USA; 2 Department of Plant and Microbial Biology, North Carolina State University, Raleigh, NC 27695, USA; ethomas@ncsu.edu (E.T.); margaret_daub@ncsu.edu (M.E.D.)

**Keywords:** black Sigatoka, cell wall, dispensable chromosomes, Dothideomycetes, effectors, genomics, host resistance, pathogenicity, secondary metabolism

## Abstract

The international importance of banana and severity of black Sigatoka disease have led to extensive investigations into the genetic characteristics and metabolic interactions between the Dothideomycete *Pseudocercospora fijiensis* and its banana host. *P. fijiensis* was shown to have a greatly expanded genome compared to other Dothideomycetes, due to the proliferation of retrotransposons. Genome analysis suggests the presence of dispensable chromosomes that may aid in fungal adaptation as well as pathogenicity. Genomic research has led to the characterization of genes and metabolic pathways involved in pathogenicity, including: secondary metabolism genes such as *PKS10-2*, genes for mitogen-activated protein kinases such as *Fus3* and *Slt2*, and genes for cell wall proteins such as glucosyl phosphatidylinositol (GPI) and glycophospholipid surface (Gas) proteins. Studies conducted on resistance mechanisms in banana have documented the role of jasmonic acid and ethylene pathways. With the development of banana transformation protocols, strategies for engineering resistance include transgenes expressing antimicrobial peptides or hydrolytic enzymes as well as host-induced gene silencing (HIGS) targeting pathogenicity genes. *Pseudocercospora fijiensis* has been identified as having high evolutionary potential, given its large genome size, ability to reproduce both sexually and asexually, and long-distance spore dispersal. Thus, multiple control measures are needed for the sustainable control of black Sigatoka disease.

## 1. Introduction

Bananas and plantains are considered one of the most important crops in developing and in tropical and subtropical countries [1,2]. World production of bananas and plantains exceeded 162 million tons in 2020, with production areas of 11.7 million hectares [3]. Bananas are important both as a subsistence crop and as an export crop: 85% or more of bananas grown are consumed locally, with the rest exported [1,4]. Bananas are the world’s most exported fresh fruit, with a value of approximately USD 10 billion/year, and in 2020, over 21 million tons of bananas were exported globally [5,6]. Current edible banana cultivars include diploids, triploids, and tetraploids of two banana species: *Musa acuminata* and *Musa balbisiana* [7]. Cavendish cultivars make up the vast majority of bananas grown for export; these are triploid “dessert” banana varieties of *M. acuminata* [8,9].

**Figure 1:**
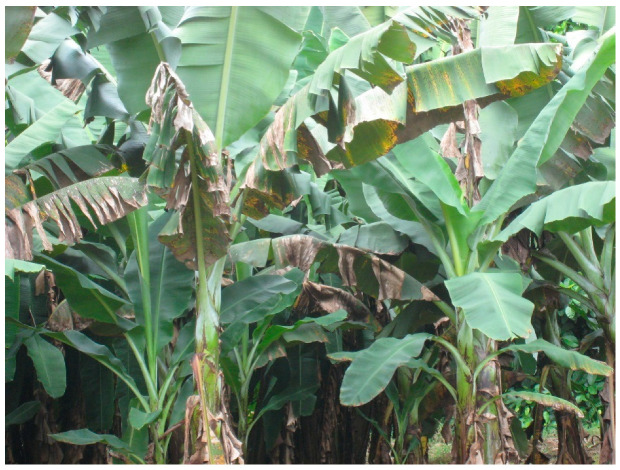
Banana plants exhibiting necrotic leaf streaks characteristic of black Sigatoka disease.

Black Sigatoka, caused by the ascomycete fungus *Pseudocercospora fijiensis*, is considered the most important banana disease [10]. This disease results in necrotic streaks on the leaves, a loss of photosynthetic capacity, and reduced yield (Figure 1) [9]. It also causes premature fruit ripening, which can be problematic for export companies, since the fruit can become over-ripe in transit [11]. Yield losses can exceed 50% in the absence of treatment [12]. Black Sigatoka was first described in the Sigatoka Valley in Fiji in 1963, although herbarium samples suggest that it was present in Taiwan by 1927 [9,10,13]. Since then, it has spread to most banana-growing regions around the world, including Latin America, Asia, Africa, and throughout the Pacific [9]. The disease’s importance has made it the subject of many extensive studies. For example, strategies for genetic improvement were identified in a study that queried over 3000 papers from six databases to identify genes, proteins, and pathways critical to the response of resistant banana cultivars [2]. Strobl and Mohan utilized climate and disease distribution data over 50 years to document the relative roles of crop importation and environmental dispersal in spread of the disease, using it as a model for the global impact of plant diseases [14].

**Figure 2:**
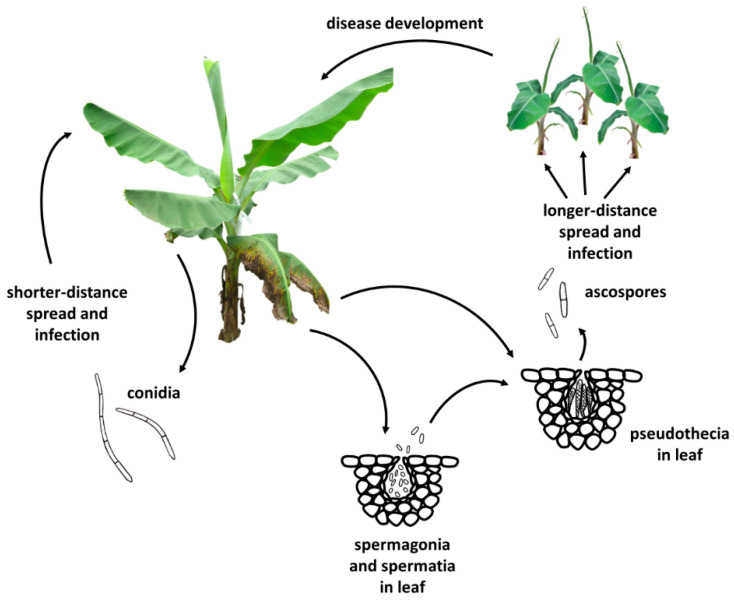
Disease cycle of black Sigatoka. *Pseudocercospora fijiensis* produces conidia on diseased leaves. Conidia are thought to be more important for short-distance spread to infect healthy tissue and generate new infections. Spermagonia in diseased leaves release spermatia which fuse with nearby receptive hyphae and develop pseudothecia. Pseudothecia release ascospores, which can spread longer distances to generate new infections [9,11].

*Pseudocercospora fijiensis* reproduces both sexually and asexually. Ascospores are considered the primary means of disease spread since they are smaller, more abundant, and can travel several kilometers via wind currents [10,11,15]. Once conidia or ascospores land on new leaves in wet or high humidity conditions, they germinate and grow epiphytically on the leaf surface before forming stomatopodia and infecting via the stomata [9,16,17]. There, the hyphae encircle the substomatal chamber and then ramify intercellularly through the leaf tissue [9,18]. Although no haustoria form, there is an extended period of biotrophy that can last for several weeks before the fungus switches to necrotrophy, at which point necrotic leaf streaks can be observed [9,19]. Hyphae in the substomatal chamber give rise to both conidiophores producing conidia and spermagonia producing the male gametes called spermatia [9,20]. Pseudothecia with ascospores are produced in necrotic leaf tissue, and ascospores are discharged upon leaf wetting, continuing the cycle (Figure 2) [21,22].

## 2. Disease Management

Disease control is accomplished on commercial plantations by fungicide treatments, which are applied up to 70 times per year and are estimated to comprise 10 to 15 percent of the total production cost of banana in Costa Rica (Miguel Muñoz, Dole-Standard Fruit Company de Costa Rica, 15 February 2022). Although the need for fungicides is an expensive problem for commercial plantations, it is especially devastating to subsistence farmers, who may not be able to afford the fungicides used to treat the disease [10]. Chemical control is achieved via a combination of site-specific and protectant/multi-site fungicides. Eight main site-specific classes of fungicides with different modes of action are commonly used in mixtures or alternating patterns: QoIs (quinone outside inhibitors), DMIs (demethylation inhibitors), SDHIs (succinate dehydrogenase inhibitors), BCMs (benzimidazoles), N-phenylcarbamates, anilinopyrimidines, amines, and guanidines [23], with DMIs the most widely used [24]. Medium to high levels of resistance have been reported in many countries for BCMs, DMIs, and QoIs, and resistance has been reported for SDHIs [9,11,23,24,25]. A recent study of resistance to DMIs in *P. fijiensis* from eight countries in South and Central America, the Caribbean, Africa, and south-east Asia identified a diversity in resistance responses to three DMI fungicides and defined the genetic basis of resistance as mutations in a gene for a cytochrome P450 enzyme (CYP51) [24]. Multi-site fungicides used for black Sigatoka control include mancozeb, chlorothalonil, metiram, captan, thiram, and propineb; these are considered fungicides of low resistance risk [23]. Additionally, biocontrol strains of bacterial and fungal species of *Bacillus*, *Melaleuca*, *Trichoderma* and *Saccharomyces*, used by themselves or in mixtures, have shown effectiveness in the control of black Sigatoka [23,26].

In addition to chemical control, cultural practices can aid in the management of black Sigatoka disease. Growing plants in good, fertile soil results in more vigorous plants with reduced disease severity [27,28,29]. Pruning necrotic leaves or leaf areas greatly reduces the amount of inoculum and therefore reduces disease spread [27]. Pruned leaves should ideally be removed from the plantation and burned or buried to eliminate the inoculum, al-though in practice this can be prohibitively labor-intensive [27]. More commonly, pruned leaves are left lying against the plantation soil, which reduces inoculum but not as well [27]. Another important cultural practice is the use of disease-free planting material. While bananas have traditionally been planted from suckers, this practice can spread a variety of diseases from field to field [30]. Therefore, large companies have turned to tissue culture to propagate vigorous plants that are free from many important pathogens [31]. Al-though wind dispersal of *P. fijiensis* ascospores is likely to quickly infect new plantings, tissue culture can help ensure that the plants are as free from the stress of other diseases as possible. Furthermore, it has long been observed that symptoms of Sigatoka diseases are much less severe when plants are grown in partial shade [9]. An early management recommendation for yellow Sigatoka was to plant in the shade of coffee trees to mitigate symptoms. This was shown to increase marketable banana yields by 50% [32]. For black Sigatoka, although the incubation period is the same regardless of lighting conditions, development of further symptoms is slowed in shaded conditions, leading to fewer necrotic leaves [33].

## 3. Breeding for Resistance

The use of resistant banana cultivars would provide an environmentally friendly means of black Sigatoka control. Active breeding efforts are ongoing at research institutes in Africa, Asia, and South and Central America [2,34]. Resistant varieties of diverse cultivars and types of bananas have been developed that are used in breeding programs and, to a limited extent, grown commercially [2,35]. Cultivated bananas are triploid, sterile, and parthenocarpic, which makes conventional breeding difficult; diploid varieties such as the *Musa acuminata* variety ‘Calcutta 4’ have served as the basis for many breeding programs [2,36,37]. Ortiz and Vuylsteke studied the genetic inheritance of resistance by crossing ‘Calcutta 4’ with susceptible triploid plantain cultivars [38]. Segregation ratios for resistance were consistent with one major recessive allele named *bs1* and two independent alleles with additive effects, named *bsr2* and *bsr3* [38]. This is similar to the genetic basis of resistance to *Pseudocercospora musae*, the causal agent of yellow Sigatoka disease of banana, which is thought to depend in part on multiple recessive alleles [38].

Although conventional breeding for resistance is difficult and labor intensive due to the sterility of edible banana cultivars, successful crosses have been achieved by international research institutes, and black-Sigatoka-resistant dessert bananas, cooking bananas, and plantain varieties have all been developed [2,34,39]. One of the difficulties faced has been the breakdown of resistance, due to the limited sources of resistance used in breeding efforts [34,40]. The problem of resistance loss led Kimunye et al. to screen banana accessions to identify additional sources of resistance [34]. Development of resistant cultivars based on diverse sources of resistance genes is needed to help manage black Sigatoka disease.

## 4. Genomic Structure of *Pseudocercospora fijiensis*

*P. fijiensis* research has been facilitated by sequencing and genome assembly of isolates CIRAD86, CIRAD 139a, and IIL-20 [41,42,43] as well as development of genetic linkage maps [41,44]. Genome analysis reveals several striking things about *P. fijiensis* compared to its relatives in the class Dothideomycetes. *P. fijiensis* has a 74-Mb genome, which is greatly expanded compared to other Dothideomycetes [41]. Its increased genome size is due to the proliferation of long terminal repeat (LTR) retrotransposons [41]. Analysis of terminal repeats of LTR retrotransposons in *P. fijiensis* suggests that the proliferation of these transposons happened relatively recently in evolutionary history [41]. It is currently unknown why LTR retrotransposon expansion has occurred so extensively in *P. fijiensis*. The gene *rid*, which is the only gene known to be required for Repeat-Induced Point (RIP) mutation, seems to be intact in the *P. fijiensis* genome, and the genome bears extensive C to T transitions which are characteristic of the RIP phenomenon [41]. Because RIP is only active during sexual reproduction [41,45], it is hypothesized that periods of asexual reproduction could explain retrotransposon proliferation [41].

Another interesting feature of the *P. fijiensis* genome is that there is a high incidence of chromosome length polymorphisms, observed both in a collection of isolates from different geographical areas in Mexico [46] and in a collection of isolates within the same field in Costa Rica [41]. This finding indicates a high variability in genome content or organization between isolates, and may reflect deletion, insertion, or translocation events [47]. In comparing the *P. fijiensis* isolate CIRAD139A to the originally sequenced strain CIRAD86, about 12% of sequencing reads did not map to the reference genome, suggesting that CIRAD139A may have additional genome sequences compared to the reference strain [41].

An increasing number of fungal pathogen genomes have been shown to contain accessory or conditionally dispensable chromosomes. These conditionally dispensable chromosomes may confer a fitness advantage in particular environments, but outside of those conditions can be lost without impacting fitness [48]. In many cases, their function is cryptic, but in some fungi, genes encoded on the dispensable chromosomes play roles in pathogenicity. For example, genes for AM-toxin, HC-toxin production, and phytoalexin detoxification are encoded on dispensable chromosomes in *Alternaria alternata*, *Cochliobolus carbonum,* and *Nectria haematococca*, respectively [49,50,51].

Most dispensable chromosomes in filamentous fungi are small and rich in repetitive sequences [48]. Codon usage and GC content often differ from the core chromosomes, suggesting that dispensable chromosomes may have originated at least in part by horizontal DNA transfer [47,52]. The related pathogen *Zymoseptoria tritici* has eight chromosomes that are dispensable and are easily lost during sexual reproduction [53]. Fourteen scaffolds from the *P. fijiensis* reference genome (isolate CIRAD86) share several characteristics with the dispensable chromosomes from *Z. tritici*: they are small, have low G+C content, different codon usage, highest proportion of repetitive DNA, lowest gene density, and lowest proportion of genes encoding proteins with PFAM domains [43,54]. Therefore, it has been hypothesized that the 14 scaffolds from *P. fijiensis* may also represent dispensable chromosomes [43,54].

Transcriptome analysis provides further support for this hypothesis. In a study comparing gene expression of the *P. fijiensis* isolate 14H1-11A in infected banana leaf tissue versus culture medium, the 14 putative dispensable scaffolds had a very different pattern of gene expression than the core scaffolds [55]. Each of the putative ‘core’ scaffolds had a small percentage of genes with higher expression in the infected leaf tissue and a small percentage of genes with higher expression in the medium [55]. On two of the putative dispensable scaffolds, a very high percentage (>30% and >50%) of the genes had higher expression in the infected leaf tissue, whereas no genes on those scaffolds had higher expression in the medium, which suggests that these two scaffolds may be important for pathogenicity [55]. For the remaining putative dispensable scaffolds, some had a small percentage of genes with higher expression in one condition but not the other, and some putative dispensable scaffolds had no differentially expressed genes [55]. Further analysis of scaffolds with no differentially expressed genes revealed that for two of them, no transcripts were detected at all [55]. PCR assays targeting genomic DNA were unable to amplify any of three genes tested from one of the scaffolds from which no transcripts were detected, and could amplify one of three genes from the other scaffold from which no transcripts were detected [55]. These results suggest that these scaffolds or large parts of them may be absent in *P. fijiensis* isolate 14H1-11A, which is consistent with the hypothesis that these scaffolds represent dispensable chromosomes. If the chromosomes corresponding to these 14 *P. fijiensis* scaffolds are indeed dispensable, they have a different evolutionary origin than the ones in *Z. tritici*, since genes on each species’ dispensable chromosomes or putative dispensable chromosomes are absent in the genome of the other species [54].

## 5. Secondary Metabolism

Many pathogens produce secondary metabolites such as polyketides and non-ribosomal peptides as important pathogenicity factors. These can have a wide variety of modes of action, but often they cause toxicity to plant cells during a necrotrophic stage of infection by the pathogen. *P. fijiensis* is closely related to species of fungi that are known to produce polyketide light-activated toxins, such as *Cercospora* spp. which produce cercosporin and *Ramularia* spp. which produce rubellin [56,57,58]. Since it has long been observed that Sigatoka disease symptoms are mitigated by growing plants in partial shade [9], it has been suspected that *P. fijiensis* may also produce light-activated toxins. Therefore, a number of experiments have been carried out to identify toxic secondary metabolites from *P. fijiensis*.

**Figure 3:**
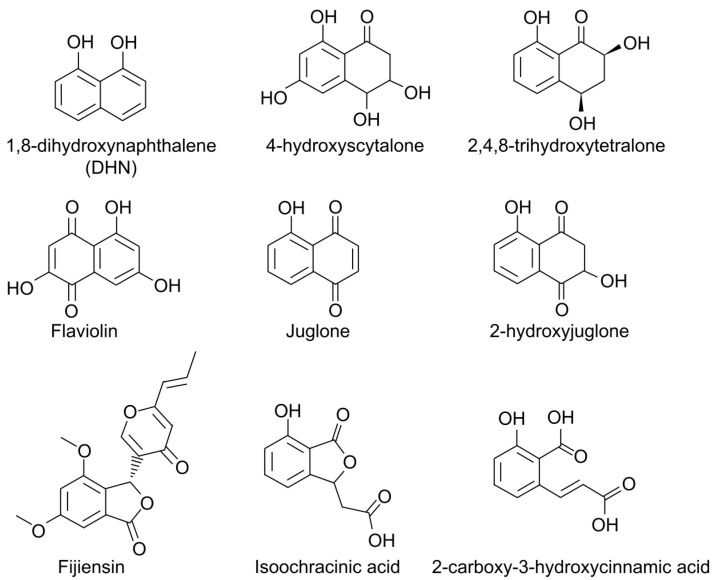
The figure shows the structures of secondary metabolites identified from *P. fijiensis* that are known to be toxic to banana tissue, as well as the structure of 1,8-dihydroxynaphthalene (DHN), which is polymerized to produce DHN melanins. Some of the phytotoxic shunt metabolites are derived from this pathway [59,60,70,71,72,73,74].

The first phytotoxic secondary metabolite from *P. fijiensis* to be reported, fijiensin (Figure 3), is produced in modified M-1-D liquid medium and was found to induce necrotic lesions on both resistant and susceptible banana cultivars but not non-host species [59,60]. Fijiensin, also called vermistatin, is produced by many other fungi, including *Talaromyces flavus* [61], *Talaromyces thailandiasis* [62], *Penicillium vermiculatum* [63,64], *Penicillium verruculosum* [65], *Penicillium simplicissimum* [66], *Eurotium rubrum* [67], and *Guignardia* sp. [68]. No fungicidal activity of fijiensin could be observed toward *Saccharomyces cerevisiae* or *Aspergillus niger* [68]. However, it does potentiate the effects of the fungicide miconazole against *Candida albicans* [61]. Compounds related to fijiensin have been shown to slightly stimulate the rooting of lettuce and Chinese cabbage seedlings [69]. In P-388 and EAC leukemia cells, it acts as an RNA synthesis inhibitor and has shown some promise as an antitumor compound [68].

Additional phytotoxic metabolites of *P. fijiensis* were identified by Stierle and colleagues from cultures grown in M-1-D medium and extracted in ethyl acetate: 2,4,8-trihydroxytetralone (2,4,8-THT), juglone, isoochracinic acid, 2-carboxy-3-hydroxycinnamic acid, and 4-hydroxyscytalone (Figure 3) [60]. Of these, 2,4,8-trihydroxytetralone, juglone, and 4-hydroxyscytalone are melanin shunt pathway metabolites [72,73]. Juglone and 2,4,8-THT were only found in *P. fijiensis* cultures older than 20 days, at which point 2,4,8-THT was produced at 30 times the rate of the other toxins. Toxicity of these metabolites was compared for resistant and susceptible banana cultivars, and only 2,4,8-THT exhibited host selectivity with this assay [60].

The fungicide tricyclazole blocks melanin biosynthesis and leads to the accumulation of melanin shunt pathway metabolites. For *P. fijiensis*, the addition of tricyclazole to the culture medium results in increased production of 2,4,8-THT and the naphthoquinone flaviolin, but not 2-hydroxyjuglone or juglone. Tricyclazole treatment of *P. fijiensis*-infected banana plants resulted in large necrotic leaf spots [70]. 2,4,8-THT production by *P. fijiensis* in culture was shown to be increased by banana leaf extracts in the medium, especially extracts from resistant compared to susceptible banana cultivars [70]. Based on these data, 2,4,8-THT was proposed as an important pathogenicity factor for black Sigatoka, and it was recommended as a screening tool to identify resistant plants in tissue culture systems.

Other studies have examined the potential role in the disease process of the melanin shunt metabolite juglone, a compound first identified as a product of plants in the family Juglandaceae [75]. In the study by Stierle and colleagues, juglone was isolated at lower concentrations but was more phytotoxic than 2,4,8-THT [60]. Busogoro and colleagues extracted fungal metabolites from culture filtrates using ethyl acetate and injected these metabolites into banana leaves. They showed toxicity that was light-dependent and that resulted in swelling of the chloroplasts [74]. Juglone inhibits electron transport in banana chloroplasts, and this inhibition is greater for the black-Sigatoka-susceptible cultivar ‘Grand Nain’ compared to the partially resistant cultivar ‘Fougamou’ [74]. This mode of action for juglone toxicity suggests an explanation for the light dependence of black Sigatoka symptoms. However, yellow Sigatoka symptoms are also alleviated by growing banana plants under shade, and Stierle and colleagues were unable to show production of juglone or 2,4,8-THT by *P. musae* in culture [60].

Melanin itself is an important pathogenicity factor for many fungi. It is crucial for penetration of cells by certain appressoria-producing fungi, including *Magnaporthe grisea* [76] and *Venturia inaequalis* [77]. For *M. grisea*, melanized appressorial cell walls allow the accumulation of glycerol and high turgor pressure in the appressoria [76]. In the human pathogen *Wangiella dermatitidis*, melanin-deficient mutants are less pathogenic, and grow much more slowly through agar medium, an effect which is thought in part to be due to reduced ability for hyphal tip protrusion [78]. Melanin also reduces cell wall porosity, which can protect the fungus against fungicides and other toxic compounds, perhaps ones produced by the host plant [79].

Melanin can scavenge and protect against reactive oxygen species (ROS). For example, melanized cells of *Cryptococcus neoformans* survive better than non-melanized cells in the presence of free radicals of oxygen and nitrogen [80]. ROS-scavenging ability is important for a pathogen, since an oxidative burst is a common host defense in both plants and animals [81,82]. In other cases, such as under UV light, melanin can actually generate ROS, including singlet oxygen, hydroxyl radicals, and hydrogen peroxide [83,84]. Isolated melanin as well as whole mycelium from *P. fijiensis* have been shown to produce singlet oxygen when irradiated with 532 nm light [85]. Production of ROS could be used to kill host tissue to obtain nutrients, a strategy reported for many necrotrophic and hemibiotrophic pathogens [86,87,88].

More recently, the polyketide synthase gene for melanin production, *PKS10-1*, was knocked out, and this knockout was reported to be as virulent as the wild-type strain [9]. Since no alternative pathways to melanin shunt metabolites have been reported, this knockout should be unable to produce melanin or melanin shunt-pathway metabolites. This study suggests that melanin and the shunt metabolites 2,4,8-THT, juglone, and 4-hydroxyscytalone may not play an important role in the disease process. However, chemical analyses of the mutant would be required to confirm its inability to produce 2,4,8-THT and the other shunt metabolites.

While no evidence of phytotoxins was found in the aqueous phase of M-1-D culture filtrates [60], multiple studies have found evidence of hydrophilic phytotoxins when *P. fijiensis* is grown in liquid V-8 medium [89,90]. For these assays, a pigment-free fraction was obtained using activated charcoal, and this fraction was shown to be more phytotoxic than the original crude filtrate [89]. Although the identity of these phytotoxins has not yet been published, one phytotoxin has been shown to have similar activity in both light and dark, and cause an increase in ROS and membrane permeability [90]. This phytotoxin was toxic to non-host species and to resistant and susceptible banana varieties, indicating that it is a non-host-selective toxin [90].

Bioinformatics analyses and transcriptomics are beginning to provide information about the secondary metabolism genes from *P. fijiensis*. Genes commonly associated with secondary metabolism, such as those encoding short-chain dehydrogenases, oxidoreductases in the 2-oxoglutarate and Fe(II)-dependent oxygenase superfamily, and cytochrome P450s have been identified and shown to have higher expression in infected leaf tissue compared to in culture [55]. Genes encoding seven polyketide synthases (PKS), one hybrid polyketide synthase/non-ribosomal peptide synthase, and thirteen non-ribosomal peptide synthases (NRPS) have also been identified from the *P. fijiensis* genome [41,91]. Secondary metabolite biosynthetic genes are typically clustered together in fungal genomes, and such gene clusters may include a backbone gene encoding a PKS, NRPS, or diterpene synthase, as well as transcription factors and transporters, and accessory enzymes such as cytochrome P450s, short-chain dehydrogenases, and others [92]. Predictions have therefore been made about which genes may be part of some secondary metabolite gene clusters [55,91].

Recent studies of predicted PKS gene clusters identified through transcriptome analysis showed that genes in the predicted *PKS7-1*, *PKS8-1*, *PKS8-2*, *Hybrid8-3*, *PKS8-4*, and *PKS10-2* gene clusters have significantly higher expression in infected leaves than they do when the fungus is grown in culture medium or relative to expression in conidia used to inoculate plants [91,93,94,95]. These results suggest that these PKS clusters may play roles in pathogenicity.

PKS7-1 is predicted to be a non-reducing enzyme. So far, no culture conditions have been identified that promote gene expression of this PKS, whereas expression of the genes in this cluster is high in infected leaf tissue [91,94]. The other Sigatoka disease complex pathogens, *P. musae* and *P. eumusae*, do not have a close homolog of PKS7-1, and a 2016 analysis was unable to identify close homologs from other members of Mycosphaerellaceae [91].

PKS8-1 is predicted to be a non-reducing enzyme, and the *PKS8-1* gene cluster has been conserved across many species within Dothideomycetes [95]. The genomes of the closely related species *P. musae*, *P. eumusae*, and *P. pini-densiflorae* contain nearly an identical combination of genes adjacent to the PKS, including those encoding an MFS transporter, a cytochrome P450, a dehydrogenase, two aldo/keto reductases, a glyoxylase/beta-lactamase, a decarboxylase, an AflR-like transcription factor, and an AflJ-like regulatory protein [95]. The conservation of these genes within *Pseudocercospora* species suggests that these species produce a very similar polyketide product. Phylogenetics analysis with characterized PKS sequences indicates that PKS8-1 forms a clade with the monodictyphenone-producing MdpG PKS from *Aspergillus nidulans* and the cladofulvin-producing ClaG PKS from *Cladosporium fulvum* [95]. The monodictyphenone, cladofulvin, and *PKS8-1* gene clusters have genes encoding some similar accessory enzymes, but have several differences as well, suggesting that while the polyketide product of the *PKS8-1* gene cluster may be similar to cladofulvin and monodictyphenone, it is not the same [95]. A time-course experiment of cluster gene expression in infected plants showed significantly increased expression of the PKS and five of the clustered genes from 2 weeks post inoculation through 9 weeks, relative to expression in the conidia used to inoculate plants [95]. To further characterize this gene cluster, an overexpression mutant of the AflR-like transcription factor was generated. Notably, cluster genes that had increased expression in infected leaf tissue compared to conidia all had increased expression in culture in an overexpression mutant of the AflR-like gene [95]. However, overexpression of the AflR-like gene was not sufficient to increase expression of the cluster genes in planta, and perhaps for that reason, no differences were observed in pathogenicity between the wild-type and overexpression mutant [95].

PKS8-2 is predicted to be a highly reducing enzyme [91]. The gene is expressed in some culture conditions but has increased expression in infected leaf tissue compared to in culture [91,94]. A time-course analysis showed significant increases in expression of the PKS and five clustered genes from 2 weeks post-inoculation through 9 weeks [94]. In a phylogenetics analysis, PKS8-2 forms a clade with the fumonisin-producing PKS from *Fusarium verticillioides* [91]. Fumonisin acts as an analog to sphinganine and thereby perturbs sphingolipid biosynthesis [96]. Both PKS8-2 and the fumonisin-producing gene cluster from *Fusarium* contain genes homologous to α-oxoamine synthase (an enzyme involved in sphingolipid biosynthesis), and the *PKS8-2* gene cluster also has genes encoding ketosphinganine reductase and sphingolipid hydroxylase-like enzymes [91]. The phylogenetics analysis of the PKS and the composition of the *PKS8-2* gene cluster suggest that the polyketide product may also act to perturb sphingolipid biosynthesis, although further research would be needed to prove this hypothesis. *PKS8-2* RNAi mutants were generated, but efficacy of silencing was poor and no consistent difference in pathogenicity was observed between the silencing mutants and the non-silenced vector control [94].

Although analysis of the above PKS clusters have yet to identify the pathway product or biological function, functions for two additional clusters (*PKS8-4* and *PKS10-2*) have been identified. PKS8-4 and the hybrid PKS-NRPS enzyme Hybrid8-3 are predicted to be partially reducing enzymes. The corresponding genes are more highly expressed in infected leaf tissue compared to culture medium. Analysis of the *PKS8-4* gene promoter using a pPKS8-4:GFP transcriptional fusion showed that *P. fijiensis PKS8-4* is expressed in spermagonia, the male reproductive structure, both in culture and in the substomatal chamber of infected leaves [93]. Phylogenetics analysis has shown that PKS8-4 and Hybrid8-3 form a clade with lovastatin, compactin, and betaenone-producing PKS sequences, as well as PKS sequences from *Sordaria macrospora* and *Neurospora crassa* [93]. Consistent with the hypothesized role for PKS8-4 in sexual reproduction, the homologous Pks-4 from *N. crassa* and its homolog Pks4 from *S. macrospora* have been shown to be important for female fertility and perithecia development [97,98]. A *P. fijiensis pks8-4* disruption mutant was not altered in growth, conidia production, or pathogenicity [93]. No differences were observed in spermagonia development in the substomatal chamber of *pks8-4* mutants [93], however; thus, further research is needed to determine if *pks8-4* disruptants are altered in spermatia development or function.

A critical role in pathogenicity was identified for the PKS10-2 cluster [94]. PKS10-2 is predicted to be a partially reducing enzyme [91]. *PKS10-2* is expressed in some conditions in culture but has greatly increased expression in infected leaf tissue compared to in culture [91,94]. In a phylogenetics analysis, PKS10-2 forms a clade with the solanapyrone-producing PKS from *Alternaria solani*, but the gene clusters are very different, especially considering that the *PKS10-2* gene cluster contains a non-ribosomal peptide synthase [91]. This finding suggests that, while the two PKS products may produce similar initial products, the final polyketides produced by these gene clusters likely differ. The other *Pseudocercospora* species *P. musae*, *P. eumusae*, *P. fuligena*, and *P. cruenta* each have homologs of *PKS10-2* with very similar gene clusters [94]. However, the *P. fuligena* NRPS has been truncated such that it does not contain all the necessary domains for function [94]. *P. fijiensis* transformants silenced for *PKS10-2* were generated. Banana plants inoculated with the silenced strains showed limited disease development with few lesions and no lesion expansion or blighting typical of black Sigatoka disease [94]. These results suggest this cluster to be a target for engineering resistant banana through strategies such as host-induced gene silencing.

In addition to the identification of polyketide synthases that may play a role in pathogenicity or reproduction and spread of *P. fijiensis*, one non-ribosomal peptide synthase gene cluster and one NRPS-like gene cluster were found to have higher expression in infected leaf tissue compared to culture medium [55]. The closest characterized homolog to the NRPS from *P. fijiensis* is a destruxin synthetase; destruxins have insecticidal and phytotoxic properties and are produced by fungal pathogens of both insects and plants [99,100,101,102,103,104,105]. There is also a diterpenoid biosynthetic gene cluster from *P. fijiensis* that has higher expression in infected leaf tissue compared to culture medium [55]. It is very similar to a gene cluster producing a fusicoccane called brassicicene C from *Alternaria brassicicola* [55]. Fusicoccanes affect plant physiology in a variety of ways, including the opening of stomata [106].

## 6. Effectors

Because pathogenic fungi secrete effector proteins into the apoplast to modulate host physiology, effectors tend to be small, secreted proteins that are cysteine-rich to provide greater stability in the apoplast through the disulfide bonds [107,108,109]. The host often evolves to recognize pathogen effectors and trigger stronger host defenses, so genes encoding effectors are typically rapidly evolving and highly variable between different species and even between strains of the same species [110]. Accordingly, some bioinformatics predictions have been made to predict putative effectors from the *P. fijiensis* genome sequence. Separate studies have predicted over 100 effectors encoded by the *P. fijiensis* genome, with 78 candidate effectors shared between the analyses [111,112]. In a transcriptome sequencing study, 30 genes encoding effector-like proteins were identified with increased expression in infected leaf tissue 6 weeks post inoculation compared to in culture medium; for approximately half of these putative effector genes, homologs were restricted to species within the Mycosphaerellaceae, which is consistent with the observation that effectors often have a restricted phylogenetic distribution [55,110]. Chang et al. predicted seven putative core effectors common to the Sigatoka complex pathogens *P. fijiensis*, *P. musae*, and *P. eumusae* [111]. Approximately half of the candidate effector genes from each Sigatoka complex pathogen could be regarded as species-specific [111].

To date, very few effectors have been characterized from *P. fijiensis*. However, it has been shown that *P. fijiensis* shares a few conserved effectors with *Cladosporium fulvum*, including Avr4, Ecp2, and Ecp6 [111,113]. In *C. fulvum*, Avr4 is a chitin-binding lectin that protects against plant chitinases [114]. The *P. fijiensis* Avr4 homolog PfAvr4 has also been shown to bind to chitin, and it is similar enough to *C. fulvum* Avr4 to trigger a hypersensitive response in tomato plants with the cognate R protein [113]. Notably, infiltration of purified PfAvr4 into the resistant banana variety ‘Calcutta 4’ results in a hypersensitive-like response, suggesting that ‘Calcutta 4’ has an R gene for this effector [41]. *C. fulvum* effector Ecp6 functions in a similar manner to Avr4: it binds to chitin, outcompeting the host receptor for chitin, and thereby prevents an immune response [115]. Ecp6 has homologs in *P. fijiensis* and many other fungi [111,116]. Transcriptome analysis of *P. musae* showed increased expression of *Ecp6* during association with the resistant banana variety ‘Calcutta 4’ relative to ‘Grand Nain’ [117]. In *C. fulvum*, Ecp2 is a virulence factor and promotes mycelial growth in infected tissue [118]. *P. fijiensis* has three homologs of the *C. fulvum* effector Ecp2. One of the *P. fijiensis* Ecp2 homologs triggers an HR response in tomato plants with the cognate R protein for *C. fulvum* Ecp2 [113]. Expression of this *P. fijiensis Ecp2* homolog also triggers necrosis in tomato that is independent of the presence of the R gene [113].

## 7. Other Pathogenicity Genes

In addition to secondary metabolites and effectors, fungi have diverse strategies for host infection and invasion which may be targets for fungicide activity or breeding for resistance. Plant pathogenic fungi secrete cell-wall-degrading enzymes that degrade cellulose, pectin, and xylan, both to invade plant tissues and to obtain nutrients. Interestingly, the genomes of *P. fijiensis* and other members of Mycosphaerellaceae have a low number of genes encoding cell-wall-degrading enzymes, relative to other Dothideomycetes [43,54]. The reason for the low number of cell-wall-degrading enzymes is uncertain but is hypothesized to help avoid detection during the initial biotrophic phase of infection [54].

In a transcriptome analysis of *P. fijiensis*, several types of genes with pathogenicity-related homologs were identified as having higher expression in infected leaf tissue, including: genes encoding proteins with Common in Fungal Extracellular Membrane (CFEM) domains, Domain of Unknown Function (DUF) 3328, and hydrophobic surface binding protein domains, as well as salicylate hydroxylase-like proteins [55]. CFEM domains uniquely occur in fungi and are common in fungal extracellular membrane proteins, including proteins with roles in fungal pathogenesis [119,120]. In *Aspergillus oryzae*, hydrophobic surface binding protein A has been shown to adsorb to a hydrophobic surface and recruit cutinase [121]. Although the function of the DUF3328 domain is uncertain, a DUF3328 domain-containing protein is involved in cyclization in the biosynthesis of the ribosomal peptide ustiloxin, which is a toxic secondary metabolite [122]. Another DUF3328 domain-containing protein is involved in cyclization of the ribosomally encoded peptide asperipin-2a [123]. Additionally, a DUF3328 domain-containing protein is required for the production of victorin, a ribosomally encoded peptide with host-selective toxicity [124]. Salicylate hydroxylase degrades salicylic acid, which is an important signaling molecule for host defense responses. Salicylate hydroxylase was also identified from an in vitro *P. fijiensis* secretome [125].

One study compared the secretomes of an isolate of *P. fijiensis* that can infect the resistant banana variety ‘Yangambi Km 5′ (referred to as the virulent strain) versus one that can only infect the susceptible ‘Grand Nain’ (the avirulent strain). This study found that the virulent strain produced increased enzymes involved in oxidative stress response, including superoxide dismutases, peroxiredoxin, and chloroperoxidase [125]. Acid trehalase was also increased in the virulent compared to the avirulent strain [125] and may also be involved in the oxidative stress response. In *Candida* spp., disruption of an acid trehalase normally found in the cell wall results in increased sensitivity to oxidative stress and disruption of the normal cell wall structure [126].

Fungal cell walls are unique because they are rich in chitin, mannans, glucans, and glycoproteins [127]. Kantun-Moreno et al. have identified and analyzed the expression of a class of mannoproteins in *P. fijiensis*, the glucosyl phosphatidylinositol (GPI) proteins [128]. GPI proteins have been reported to function in cell wall remodeling, adhesion to host tissue, biofilm formation, and as virulence factors [129,130,131]. The *P. fijiensis* GPI proteins MfGas1 and MfGas2 are homologous to Gas proteins (glycophospholipid surface proteins) from pathogenic *Fusarium* species [128]. In *Fusarium* spp., these Gas proteins are involved in pathogenesis, cell wall formation, morphology, and conidiation [130,132]. Expression of *MfGas1* is nearly constant throughout different culture conditions and the infection process, whereas *MfGas2* is more highly expressed in conidia compared to mycelium, and during the infection process it is most highly expressed at the speck stage [128].

A study conducted by Burgos-Canul et al. compared the cell wall proteome of two isolates of *P. fijiensis* that differed in virulence. Of the 90 cell wall proteins identified and shared by both isolates, 24 were pathogenicity factors including 1,3-β-glucanase, isocitrate lyases, superoxide dismutase, peptidyl-prolyl cis-trans isomerase, as well as the fungal effectors Avr4 and Ecp6. Of the 21 unique cell wall proteins found in the highly virulent strain, 16 were virulence factors, whereas of the 39 unique cell wall proteins found in the less virulent strain, only 11 were virulence factors. In each of the isolates, many of the unique proteins detected pertained to carbohydrate metabolism as well as energy production. However, the authors found that the percentage of proteins involved in metabolic processes was higher in the less virulent strain, unlike other studies involving *Fusarium oxysporum* race 4 that found the percentage of proteins was higher in the more virulent strains [133]. In addition, acid trehalase located in the cell wall was uniquely found in the highly virulent strain [134].

In addition to proteins located in the cell wall, the membrane-bound ATP-binding cassette (ABC) transporters may play a critical role in virulence. An ABC transporter, MgAtr4, was identified in the closely related fungus *Z. tritici*. This transporter was shown to contribute to virulence and to aid in colonization of the substomatal cavities [135]. Later, a homolog of *MgAtr4* was identified in the *P. fijiensis* genome. This homolog, *MfAtr4*, was most highly expressed in early stages of infection, and it was not expressed at the latest necrotrophic phase of infection [136]. The identity of the substance being transported has not yet been determined, but ABC transporters may play several roles in virulence. They can protect the fungus against plant defense compounds, or they may transport effectors or secondary metabolites.

Mitogen-activated protein kinases (MAPKs) have been shown to be important for the virulence of *P. fijiensis*. Silenced mutants of the MAPK genes *PfHog1*, *PfFus3*, and *PfSlt2* caused delayed disease development when inoculated onto banana plants, compared to inoculation with the wild type [137,138]. The *PfHog1-*, *PfFus3-*, and *PfSlt2-*silenced mutants displayed impaired invasive growth in the banana leaf tissue after inoculation, resulting in delayed symptoms of black Sigatoka [137,138], consistent with the role of these genes in other species since homologs are known to be important for pathogenicity [139]. *PfHog1*, like its homologs in other species, was also shown to be important for adaptation to hyperosmotic conditions [137,140].

## 8. Host Defense Genes

The development of an effective control of black Sigatoka depends not only on a thorough understanding of *P. fijiensis* pathogenicity mechanisms, but also depends on understanding the resistance mechanisms in resistant banana cultivars and genotypes. Several *Musa* genomes have been published, including that of *M. acuminata* ssp. *malaccensis* ‘DH-Pahang’, *M. acuminata* ssp. *burmannica* ‘Calcutta 4’, *M. acuminata* ssp. *banksii* ‘Banksii’, *M. acuminata* ssp. *zebrina* ‘Maia Oa’, *M. balbisiana* ‘Pisang Klutuk Wulung’, *Musa itinerans*, and *Musa schizocarpa* [141,142,143,144,145].

Having the genome sequences of both banana and fungus enables a better analysis of transcriptomics data; therefore, predictions can be made of genes involved in pathogenicity and resistance to disease. Several studies have analyzed expression of banana genes during infection with *P. fijiensis*. Rodriguez et al. used microarray analysis to compare genes expressed in the susceptible ‘Williams’ variety to the resistant ‘Calcutta 4’ variety, when exposed to *P. fijiensis* [146]. ‘Calcutta 4’ showed a higher expression of genes encoding phenylalanine ammonia lyase, peroxidase, disease resistance response 1, PR-4 and PR-10 when compared to ‘Williams,’ between 6 and 24 h post inoculation [146]. Subsequent work, using gene expression and metabolite analysis, further documented a role for jasmonic acid and ethylene pathways in resistance [147]. Timm et al. generated a suppression-subtractive cDNA library of ‘Calcutta-4’ at different time points in the biotrophic phase compared to uninoculated ‘Calcutta-4’ [148]. Genes upregulated after being challenged with *P. fijiensis* include ones encoding putative transcription factors, the NBS-LRR protein RGA1, and cytochrome P450 pathways [148]. They also detected effects on genes involved in the glycolysis/gluconeogenesis pathway and scavenging of ROS [148]. Portal et al. generated a suppression subtractive hybridization cDNA library to identify transcripts that are upregulated during stage 3 (streaks become wider and change from reddish to dark brown) and stage 4 (dark brown to black spots) of the black Sigatoka disease process with the susceptible ‘Grand Nain’ banana cultivar [149]. Banana genes with antifungal action; genes for phenylpropanoid synthesis, detoxification, flavonoid, and isoflavone-related genes; and genes for synthesis of ethylene and activation of jasmonic acid were expressed [149]. Unfortunately, because the RNA extraction protocol was not well suited to *P. fijiensis*, the only fungal transcript identified was one encoding UDP-glucose pyrophosphorylase enzyme, which is involved in carbohydrate metabolism [149].

Transcriptome sequencing studies have also been used to characterize expression of banana genes after being challenged by close relatives of *P. fijiensis*. Passos et al. used 454 sequencing to compare transcripts between the resistant ‘Calcutta 4’ and susceptible ‘Grand Nain’ varieties of banana after being challenged with *P. musae* [117]. Genes upregulated in ‘Calcutta 4’ compared to ‘Grand Nain’ included numerous transcription factors, as well as genes potentially related to detoxification such as superoxide dismutases, glutathione-S-transferases, and metallothionen-like proteins, which protect cells against damage from ROS [117]. Gene transcripts for phenylalanine ammonia-lyase (PAL) were abundant, and these promote the synthesis of phytoalexins and salicylic acid [117]. An enzyme involved in production of flavonoids, 4-coumarate-CoA ligase, was also upregulated in ‘Calcutta 4’ [117]. Uma et al. used Illumina transcriptome sequencing of the susceptible ‘Grand Nain’ versus resistant ‘Manoranjitham’ banana varieties after being challenged with *P. eumusae* [150]. They observed very similar expression patterns as those found in other studies. For example, they observed a higher expression of flavonoid-producing genes and genes involved in scavenging ROS (glutathione S-transferase and peroxidase) in the resistant variety [150]. However, they also observed expression of genes in the resistant variety that may be involved in production of ROS (polyphenol oxidase and hexokinase-3). Furthermore, they showed that genes in the phenylpropanoid and other secondary metabolite pathways had higher expression in the resistant banana variety [150].

## 9. Potential for Transgenic Banana Plants Resistant to Black Sigatoka

The many challenges in banana breeding programs argue for transgenic approaches for the development of resistant cultivars. Transformation protocols for banana have been developed [151,152,153], and a better understanding of *P. fijiensis* pathogenicity genes may help identify potential transgenes to engineer black Sigatoka resistance in banana. Genetic modification can be performed in an agronomically desirable genetic background, which would minimize problems that could otherwise arise from conventional breeding with resistant but agronomically undesirable banana varieties. Unlike some other crops, concerns of traits spreading to other farmed bananas or their wild counterparts should be minimal due to the sterility of cultivated banana.

There are several promising options available for the choice of transgene. One option are antimicrobial peptides (AMPs), which are small, cysteine-rich peptides with broad toxicity against fungi and bacteria. Transgenic banana plants producing an analogue of the AMP magainin had resistance to both *P. fijiensis* and *F. oxysporum* f.sp. *cubense* [154]. Hundreds of transgenic *Musa* sp. lines have been developed to express different AMPs, and some of these lines showed resistance under in vitro conditions to another banana pathogen, *Colletotrichum musae*, but further research is needed to show whether these transgenic lines are resistant to black Sigatoka and other diseases [155,156,157]. Genes encoding hydrolytic enzymes such as chitinases have also been used. Transgenic ‘Gros Michel’ banana plants expressing a rice chitinase gene developed lesions from black Sigatoka more slowly than the non-transformed control plants [158]. One interesting strategy that has been tested in tobacco is to use a pathogen-inducible promoter to drive the expression of an elicitor. The elicitor is then recognized by the plant and triggers HR, leading to resistance [159].

Host-induced gene silencing (HIGS) is another potential strategy for engineering disease resistance. For this strategy, an RNAi-silencing construct is designed against an important fungal gene, and this construct is transformed into the plant host. When the plant expresses this construct, dsRNAs are transported from the plant to the pathogen, where they silence the pathogen genes. HIGS is reported to be effective not only for biotrophic pathogens such as powdery mildew [160] and rust fungi [161], but also hemibiotrophic pathogens such as *Fusarium verticillioides* [162], and necrotrophic pathogens such as *Sclerotinia sclerotiorum* [163]. A HIGS-based strategy was successfully used to develop resistance to *Cercospora nicotianae* in tobacco by silencing the polyketide synthase in the metabolic pathway for the toxin cercosporin [164]. The PKS genes *PKS8-4* and *PKS10-2* in *P. fijiensis* are attractive choices for using HIGS in the effort to minimize inoculum production as well as colonization and symptom development in banana.

The use of HIGS does require an intact silencing pathway in the fungus being targeted, and this pathway is not ubiquitous among fungi. For example, *Saccharomyces cerevisiae*, *Ustilago maydis*, and *Cryptococcus gattii* lack intact silencing pathways [165,166,167], indicating that this pathway has been lost multiple times during fungal evolution [165]. Fortunately, multiple studies have documented that the silencing pathway appears to be intact in *P. fijiensis*, supporting HIGS as a plausible control strategy for black Sigatoka. One study showed that dsRNAs for adenylate cyclase and DNA polymerase were able to inhibit *P. fijiensis* spore germination in vitro [168]. In addition, mutants of *P. fijiensis* have been successfully generated through transformation with silencing constructs. In the previously described studies of the role of MAP kinases in *P. fijiensis* growth, virulence, and osmotic stress adaptation, a 358 bp region of the *PfFus3* gene, a 264 bp region of the *PfSlt2* gene, and a 245 bp region of the *PfHog1* gene were used to generate silencing constructs that successfully silenced the genes when transformed into the fungus [138]. Similarly, a silencing strategy was used to generate silencing constructs of the *PKS8-2* and *PKS10-2* genes from *P. fijiensis* to confirm the role of these PKS clusters in disease [94]. In this study, a 799 bp region of the ketosynthase domain of the *PKS8-2* gene, and a 436 bp region of the ketosynthase domain of the *PKS10-2* gene were used to generate silencing constructs, and silenced transformants were generated for each gene. The inoculation of banana with silenced transformants clearly documented the role of PKS10-2 in disease development. The role of PKS8-2 was less clear, however, as silencing was less efficient for *PKS8-2*, and reduction in disease did not correlate with the degree of silencing in different transformants. The lack of a strong effect of *PKS8-2* silencing on disease development may have been due to a less essential role in pathogenicity played by PKS8-2, or it may reflect differences in silencing efficiency of the two genes. Silencing efficiency has been shown to depend on the gene being targeted as well as the length of the targeted region [169].

There are several major obstacles to the development and adoption of transgenic resistant banana plants. Development and regulatory testing of a new genetically modified (GM) crop is very expensive. From 2008 to 2012, the mean cost of bringing a new GM trait to market was USD 136 million [170]. Furthermore, there are significant hurdles in public opinion where GM crops are concerned. Much of the export banana crop is exported to Europe, and consumer acceptance of GM crops in Europe is particularly low [3,171,172].

One non-transgenic approach with much of the functionality of HIGS is to use spray-induced gene silencing (SIGS), wherein dsRNA sprays are used as a pesticide to target and knock down the expression of important pathogen genes. The persistence of dsRNAs sprayed on leaf surfaces is a major challenge and depends strongly on the formulation, but in some cases the dsRNAs can retain biological activity for over 4 weeks [173,174]. The technology has shown some promise for insect pests as well as viral, fungal, and oomycete pathogens, although research with fungal pathogens is still in the preliminary stages and dsRNA uptake varies among fungi [173,174,175].

Additionally, CRISPR/CAS9 gene editing protocols have been developed in banana [176,177]; gene editing techniques that do not introduce a transgene may be more acceptable to consumers and government regulatory agencies compared to genetically modified crops. Gene editing has been used to develop various crops (including apple, citrus, rice, tomato, cotton, wheat, and others) that are resistant to fungal, viral, and bacterial diseases [178].

Regardless of the type of control methods chosen, some thought should be given to likelihood of the fungus evolving to overcome the methods used. Achieving durable control depends on understanding the biology of the pathogen and its potential to evolve. A pathogen has higher evolutionary potential if it has a high mutation rate, a large population size, both asexual and sexual reproduction, and propagules that can spread long distances [179]. Unfortunately, *P. fijiensis* has many of these risk factors for high evolutionary potential. It has a greatly expanded genome, even compared to other members of Mycosphaerellaceae, due to transposable elements. The abundance of transposable elements would contribute to a higher mutation rate. It produces abundant spores through both sexual and asexual reproduction, and the sexual spores can be dispersed by wind for long distances. There are a few strategies available to mitigate these risk factors. If overcoming host resistance requires a large fitness penalty onto the fungus, then the evolution of new virulent races is less likely. Furthermore, multiple banana varieties with different resistance genes could be mixed in the same field, or different resistance genes could be stacked in the same variety, so that there is not one large, single selection pressure on the fungus to evolve. Since *P. fijiensis* has such a high evolutionary potential, many control measures should be used in parallel, ideally combining resistant banana varieties, chemical control methods, and cultural control methods in a way that is as economical and environmentally friendly as possible.

## 10. Conclusions and Future Directions

*Pseudocercospora fijiensis* is a major threat to banana production and studies are underway to determine the molecular basis by which this fungus from the class Dothideomycetes causes black Sigatoka disease. Fungal adaptation is aided by the significantly expanded genome of *P. fijiensis* as well as by the transposable elements and dispensable chromosomes it likely possesses. The pathogen reproduces both sexually and asexually, causes extensive yield losses, and is currently controlled by an intense regimen of fungicides that is of concern both for economic as well as environmental reasons. Although international breeding efforts have led to the development of resistant varieties that are grown both commercially as well as for breeding programs, the Cavendish subgroup, cultivars from which dominate the global commercial market, is not resistant. Strategies for improved control include improved targets for chemical control such as the fungal cell wall, expanded knowledge and characterization of genes and resistance mechanisms of diverse resistant banana genotypes, and engineering resistance in economically valuable but susceptible cultivars. Recently identified key genes that play a vital role in aiding the filamentous growth, sexual and asexual reproduction, host colonization, as well as symptom development are believed to be potential transgene choices. Using a HIGS-based approach, these transgenes can aid in the development of resistant banana cultivars. The development of resistance, along with cultural practices and chemical control, are needed for the effective control of black Sigatoka disease.

## Data Availability

Not applicable.
